# Hyperemesis Gravidarum With Paraparesis and Tetany

**DOI:** 10.7759/cureus.17014

**Published:** 2021-08-09

**Authors:** Jyotsnaa Muralitharan, Vijayakumar Nagarajan, Umarani Ravichandran

**Affiliations:** 1 Internal Medicine, Rajah Muthiah Medical College & Hospital, Chidambaram, IND

**Keywords:** hyperemesis gravidarum, subacute-onset muscle weakness, hypokalemic periodic paralysis, tetany, hypocalcemia, hypomagnesemia, paraparesis

## Abstract

Subacute-onset muscle weakness can result from channelopathies, inflammatory myopathies, thyroid dysfunction, hypoparathyroidism, vitamin D deficiency, and dyselectrolytemias like hypokalemia, hypocalcemia, and hypomagnesemia. We report a curious and extremely rare case of a 29-year-old woman with hyperemesis gravidarum presenting with disabling muscle weakness involving her lower limbs and trunk, and concurrent features of tetany. Following voluminous vomiting over the last two months, she presented with history of weakness of her lower limbs of 14 days duration, resulting in difficulty in her getting out of bed or walking unassisted. On examination, she was hypotensive (80/60 mmHg) and tachycardic (110 bpm), with flaccid weakness of her lower limbs (proximal weakness more than distal weakness - power of 1/5 at the hips bilaterally, and 3/5 at the knees and ankles bilaterally) and diminished deep tendon reflexes. She also had positive Trousseau’s sign and Chvostek’s sign. Interestingly, she also had thinned-out bluish sclerae, a high-arched palate, short stature, and bilateral conductive hearing loss. Laboratory evaluation revealed anemia, hyponatremia, hypokalemia, hypomagnesemia, hypochloremia, hypophosphatemia, and low vitamin D levels. Electrocardiogram showed prolonged QT interval. Her thyroid function test and parathyroid levels were normal. With parenteral replenishment of the electrolytes and vitamin D, her power improved and she was discharged on oral supplements. Thus, this case report demonstrates the importance of aggressive, early, and adequate management of hyperemesis gravidarum to prevent dyselectrolytemia-associated paraparesis.

## Introduction

Subacute onset of muscle weakness can result from channelopathies, inflammatory myopathy, thyroid dysfunction, hypoparathyroidism, vitamin D deficiency, and electrolyte disturbances among other causes. Electrolyte disturbances that could cause muscle weakness include hypokalemia, hypocalcemia, and hypomagnesemia. These deficiencies could occur from gastrointestinal loss, renal disorders (salt-losing nephropathies), channelopathies, and other rarer causes. 

In this case report, we present a young woman with hyperemesis gravidarum who presented with subacute-onset muscle weakness involving her lower limbs and trunk and signs of tetany.

## Case presentation

A 29-year-old Indian woman, G2 P1 L1, at 18 weeks of gestational age, presented to the Obstetrics and Gynecology Department (OBG) of our hospital in South India with history suggestive of hyperemesis gravidarum, with 12 to 15 episodes of vomiting daily for the last two months, and history of symmetrical weakness of lower limbs for the last 14 days. An Internal Medicine consultation was sought soon after admission.

The weakness was of insidious onset and progressive, resulting in difficulty getting up from sitting and squatting position, difficulty climbing stairs, progressing to difficulty in walking without support for the last seven days. There was history of difficulty in turning over in bed for the last 10 days. She had no history of weakness of the upper limbs or neck. There was no history of diurnal variation of the weakness, band-like sensation, radicular or funicular pain. She had no history of fever or trauma. She reported passing reduced quantity of high-colored urine for the last few days.

No history of similar complaints in family members was elicited. She had history of hearing impairment since childhood and has worn spectacles from a young age. Her previous pregnancy was uneventful and her three-year-old son has no similar symptoms.

On examination, the patient was conscious, oriented, and cooperative. She appeared pale, malnourished, and dehydrated. Her vitals were pulse rate: 110/min, blood pressure: 80/60 mmHg, respiratory rate: 14/min, and temperature: 98.4℉. Examination of the cardiovascular system was normal. Her lungs were clear to auscultation. On abdominal examination, uterus was of 16 to 18 weeks gestational size with normally palpable fetal movements and fetal parts.

On examination of the nervous system, her higher mental functions and cranial nerves were intact. Spinomotor system examination revealed generalized muscle wasting and the lower limbs were in an attitude of abduction and external rotation at the hips, flexion and external rotation at the knees, with the ankles in neutral position. Findings of hypotonia of bilateral lower limbs with reduced power (proximal > distal) (Table 1) and diminished deep tendon reflexes were elicited.

**Table 1:** Spinomotor system examination - muscle power

	Right	Left
Hip	1/5	1/5
Knee	3/5	3/5
Ankle	3/5	3/5
Shoulder	4+/5	4+/5
Elbow	5/5	5/5
Wrist	5/5	5/5
Hand grip	90%	90%

The upper limbs showed normal tone, power (Table 1), and reflexes. Bilateral flexor plantar response was present. Examination of the sensory system, spine, cranium, and cerebellum was normal. There was neither muscle tenderness nor skin rashes present to suggest an inflammatory myopathy.

**Figure 1:**
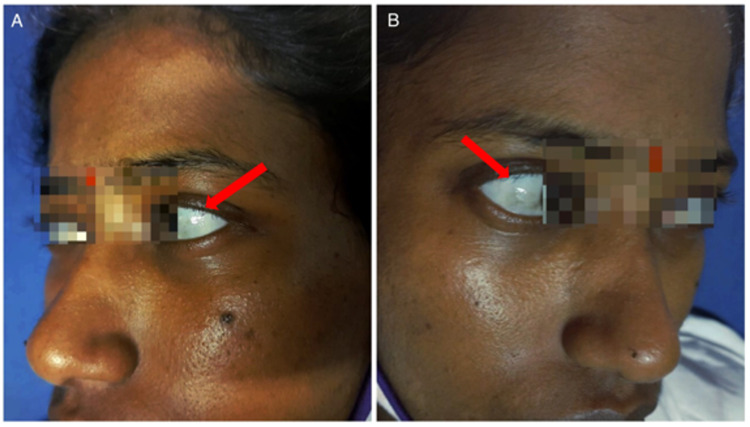
Bilateral thinned-out sclerae with bluish hue

**Figure 2:**
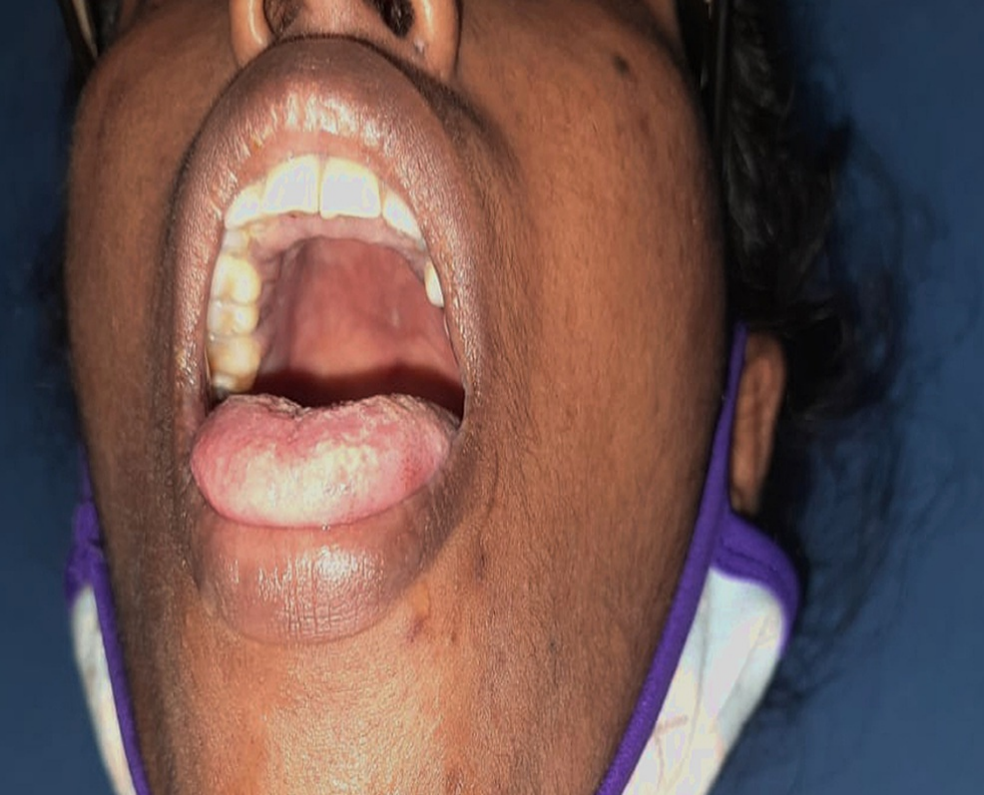
High-arched palate

**Figure 3:**
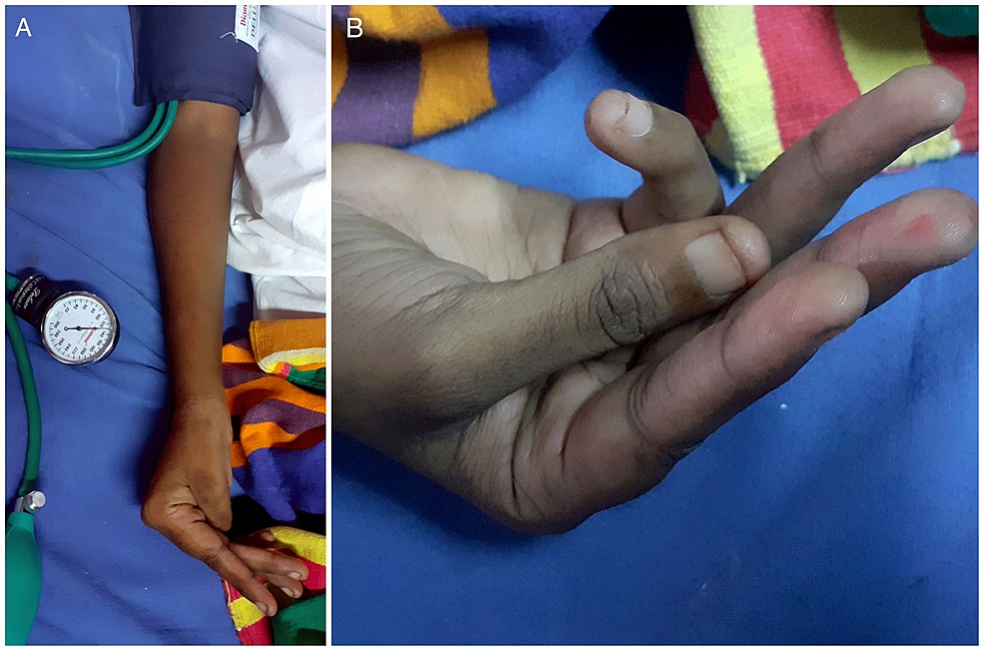
Positive Trousseau’s sign indicative of hypocalcemia

She was seen to have thinned-out sclera bilaterally with a bluish hue (Figure 1A, 1B). Oral examination revealed a few pits on her incisors, high-arched palate (Figure 2), and oropharyngeal candidiasis. She was of short stature. There was no skin laxity or hypermobility of joints. Gorlin sign, wrist sign (Walker-Murdoch sign), and thumb sign were negative. Other features of a Marfanoid habitus were lacking. She had positive Trousseau’s sign (Figure 3A, 3B) and positive Chvostek’s sign indicative of tetany. Tuning fork tests showed features of bilateral conductive hearing loss, worse in the left ear - Rinne’s test showed bone conduction >air conduction bilaterally and Weber’s test lateralizing to the left ear.

**Figure 4:**
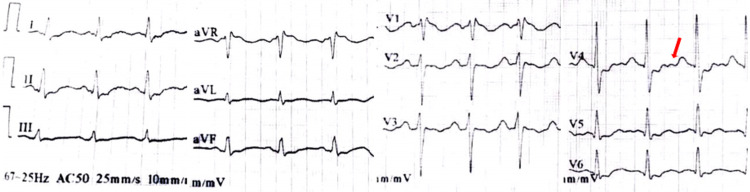
Electrocardiogram showing prolongation of QT interval (corrected QT interval of 536 msec) and U waves (red arrow)

Blood investigations showed moderate anemia (hemoglobin: 6 g/dl) of normocytic normochromic type, hyponatremia (106 mg/dl), hypokalemia (2.7 mg/dl), hypochloremia (93 mg/dl), hypocalcemia (8.3 mg/dl), hypomagnesemia (1.5 mg/dl), and hypophosphatemia (2.9 mg/dl). Arterial blood gas analysis was normal. There was no significant loss of electrolytes in urine. She had low vitamin D levels (15 ng/ml) with normal parathyroid hormone (PTH) levels. Her thyroid function and creatine phosphokinase were within normal limits. Electrocardiogram (ECG) showed a prolonged QT interval (corrected QT interval of 536 msec) and U waves (Figure 4); an echocardiogram done was normal. Urea and creatinine were indicative of a pre-renal acute kidney injury (AKI). Electromyography could not be done as the patient and her husband did not consent for the same.

The patient was managed with intravenous (IV) fluids, 3% sodium chloride infusion, IV potassium chloride infusion, oral magnesium and phosphate supplementation, IV calcium correction, promethazine, oral doxylamine, and vitamin D3. Packed cells were transfused for anemia, followed by oral hematinics. Dermatology, ENT, and Ophthalmology consultations were obtained, with OBG follow-up.

There was moderate improvement in weakness with the above treatment. Her pre-renal AKI was reverted. However, hypokalemia was persistent. Power in the hips improved to 2/5 and in the knees and ankles to 4/5. The patient was discharged on oral potassium, magnesium, and calcium supplementation and was taught physiotherapy exercises to continue. The patient was advised to review for follow-up after two weeks.

## Discussion

Our patient, a 29-year-old antenatal woman, had presented with disabling symmetrical weakness of lower limbs and trunk of subacute onset (14 days duration) following untreated severe hyperemesis gravidarum over the last two months. On neurological examination, she was found to have bilateral lower limb weakness (proximal weakness greater than distal) with diminished deep tendon reflexes, decreased power of trunk muscles with normal power and reflexes in the upper limbs. There were features of negative fluid balance (hypotension, high-colored urine) consistent with volume loss. Laboratory investigations supporting fluid and electrolyte loss in this patient included hyponatremia (106 mg/dl), hypokalemia (2.7 mg/dl), hypochloremia (93 mg/dl), hypocalcemia (8.3 mg/dl), hypomagnesemia (1.5 mg/dl), hypophosphatemia (2.9 mg/dl), and elevated serum urea and creatinine. In addition, examination findings of positive Trousseau's sign and positive Chvostek's sign indicative of tetany were present in our patient. Urinalysis did not reveal significant loss of electrolytes in urine. Arterial blood gas analysis was normal. Her ECG showed a significantly prolonged QT interval (corrected QT interval of 536 msec) and U waves.

Considering the clinical presentation of the patient and the above findings, the most appropriate diagnosis for her muscle weakness and tetany is the significant gastrointestinal loss of electrolytes from inadequately managed hyperemesis gravidarum. Hypokalemia, hypocalcemia, and hypomagnesemia resulting from electrolyte loss have manifested as flaccid weakness involving the lower limbs and trunk muscles with diminished deep tendon reflexes. These electrolyte disturbances are also responsible for the ECG findings. Prolonged QT interval with reduction of QT interval following calcium, potassium, and magnesium correction favors the same. The prolongation of QT interval is, therefore, attributable to the cumulative effect of hypocalcemia, hypokalemia, and hypomagnesemia. The U waves on ECG are consistent with hypokalemia. Tetany is most likely the result of the compounded effect of hypocalcemia and hypomagnesemia, as our patient had only mild hypocalcemia (8.3 mg/dl) and hypomagnesemia (1.5 mg/dl) on blood testing, each of which, by themselves, are not significant enough to cause the tetany features. Concurrent loss of fluid has resulted in a negative fluid balance as evidenced by concentrated, high-colored urine, hypotension, and hyponatremia. Urea and creatinine levels improved following fluid resuscitation, indicative of a pre-renal AKI due to intravascular volume reduction. Improvement in muscle power following correction of the dyselectrolytemia further supports this diagnosis. There has been a reported case of hypokalemia occurring with hyperemesis gravidarum [1]. An isolated case of paraparesis occurring due to hypokalemia in pregnancy has also been reported [2]. However, a case such as the present case, with hyperemesis resulting in significant dyselectrolytemia (hypokalemia, hypocalcemia, hypomagnesemia), manifesting as paraparesis and trunk weakness, tetany, and prolonged QT interval on ECG, has not been previously reported.

Our patient also had low vitamin D levels (15 ng/ml). Hypovitaminosis D is known to be a cause of proximal muscle weakness. Hence, hypovitaminosis D could be a contributory factor in this patient’s muscle weakness, which was greater in the proximal lower limb muscles than distally. Despite low calcium and vitamin D levels, the PTH level was normal in this patient. Additionally, gastrointestinal loss is the more obvious cause of calcium and vitamin D deficit in this patient rather than a disorder of PTH. The treatment also included vitamin D3, following which the muscle weakness improved.

The patient had blue thinned-out sclerae and had required prescription glasses from a young age. She had a history of hearing impairment from childhood, with bilateral conductive hearing loss on examination. She was also found to have a short stature and a high-arched palate on examination. Although these seem to point toward a connective tissue disorder, there was lack of history of any similar complaints in family members. There was also neither skin laxity nor hypermobility of joints. Gorlin sign, wrist sign (Walker-Murdoch sign), and thumb sign were negative. She also did not possess any other features of a Marfanoid habitus. Additionally, she had a more obvious secondary cause for her muscle weakness and tetany, i.e., electrolyte loss via hyperemesis. Therefore, although a connective tissue disorder may have been present in this patient, and may have been coexistent with a renal tubular disorder resulting in electrolyte loss, the gastrointestinal loss of electrolytes seems more significant. More invasive investigations or genetic analysis would have been required to be performed to identify such a disorder.

The other differential diagnoses we considered in this patient were thyroid disorder-related muscle weakness and inflammatory myopathy. These were ruled out by the normal thyroid function, normal creatine phosphokinase levels, and absence of muscle tenderness or skin findings on examination.

Inherited channelopathy of the calcium or sodium channels present in skeletal muscles (i.e., hypokalemic periodic paralysis) may have also been the reason for the flaccid muscle weakness in this 29-year-old patient, which could have been precipitated by the state of stress. However, there was no spontaneous resolution of the weakness. In addition, the absence of a history of similar episodes in the past draws us away from this diagnosis and toward a secondary cause for hypokalemia.

Another rare disorder to be considered in the differential diagnosis is Anderson-Tawil syndrome. It is a rare genetic disorder occurring most often due to mutations in the KCNJ2 gene and may be either inherited by autosomal-dominant mode or occur sporadically. It is characterized by the triad of muscle weakness or paralysis (periodic paralysis), arrhythmias, and distinctive physical features. Prolonged QT interval as observed on this patient’s ECG has been described as a classic feature in Anderson-Tawil syndrome. It is also known to be associated with a prolonged QU interval, and can result in arrhythmias like ventricular tachycardia. Low potassium levels are a frequent finding in this syndrome, as is periodic weakness of the muscles, most often affecting the legs, as in this patient. Although our patient had a high-arched palate and short stature, she lacked the many distinctive dysmorphic features of Anderson-Tawil syndrome and she did not experience any arrhythmia. However, she had hypocalcemia, hypokalemia, and hypomagnesemia, which are more likely to be the cause of muscle weakness and QT interval prolongation on ECG in this patient.

An even more rare possibility to consider in the differential diagnosis is a variant of Gitelman’s syndrome. The renal tubular defect in Gitelman’s syndrome, an autosomal recessively inherited disorder occurring due to defective sodium-chloride symporter in the distal convoluted tubule of the kidney, is associated with hypokalemia, hypomagnesemia, hypocalciuria, and metabolic alkalosis. Although not typically occurring, hypocalcemic tetany in Gitelman’s syndrome has been reported in the literature as a very rare association. There have been reported cases of periodic paralysis and hypocalcemic tetany occurring in association with Gitelman’s syndrome [3-5], where hypocalcemia has been attributed to chronic hypomagnesemia resulting in altered metabolism of PTH. Although our patient had hypomagnesemia, hypokalemia, and hypocalcemia, metabolic alkalosis or wasting of salts in urine was not present. Hence, a Gitelman’s syndrome variant as the cause could be ruled out.

Salt-losing nephropathies should also be considered as possible causes of electrolyte loss. These include Bartter’s syndrome, Fanconi syndrome, and pseudohypoaldosteronism type 1. Bartter’s syndrome, which occurs due to defect in the thick ascending limb of the Loop of Henle, presents with polyuria, polydipsia, chloride-resistant metabolic alkalosis, hypokalemia, low-to-normal serum magnesium, hypercalciuria, and nephrocalcinosis. Fanconi syndrome manifests as renal tubular acidosis in addition to hypokalemia, hyponatremia, hypochloremia, growth retardation, and rickets. Pseudohypoaldosteronism type 1 results in hyponatremia, hyperkalemia, metabolic acidosis, and elevated plasma renin and aldosterone levels and is more likely to present in neonates. The presentation, physical examination findings, and laboratory findings in our patient do not correspond to any of these salt-losing nephropathies, which may, hence, be ruled out.

Electromyography, had it been done, which could not be performed in this patient due to dissenting patient and attendant, would have aided in the process of narrowing down to a diagnosis.

Therefore, the muscle weakness associated with electrolyte disturbances in this patient could be attributed to gastrointestinal loss from hyperemesis gravidarum. Vitamin D deficiency may be a contributing factor to the proximal muscle weakness. Anderson-Tawil syndrome and a connective tissue disorder cannot also be ruled out in the current case.

## Conclusions

This rare case report of a 29-year-old short-statured woman with hyperemesis gravidarum, presenting with subacute-onset weakness of lower limbs and trunk that necessitated support to get out of bed or walk, and having blue sclerae, bilateral conductive hearing loss, bilateral lower limb weakness (proximal weakness greater than distal) with diminished deep tendon reflexes, weakness of trunk muscles, features of negative fluid balance (hypotension, high-colored urine), features of tetany (positive Trousseau's sign and Chvostek's sign), prolonged QT interval on ECG, hypokalemia, hypocalcemia, hypomagnesemia, low vitamin D levels in the presence of normal PTH levels, and normal thyroid function test, on examination and investigations, highlights the importance of timely and adequate management of hyperemesis gravidarum. Failure to do so has resulted in disabling muscle weakness and ECG changes from hypokalemia, hypomagnesemia, hypocalcemia, and concurrent vitamin D deficiency, and tetany from hypocalcemia and hypomagnesemia.

In evaluating the cause of electrolyte deficiencies resulting in muscle weakness, other differential diagnoses to be considered include vitamin D deficiency, Gitelman’s syndrome (variant), Anderson-Tawil syndrome, channelopathy, and other salt-losing nephropathies.
